# Compressed Sensing of Vibration Signal for Fault Diagnosis of Bearings, Gears, and Propellers Under Speed Variation Conditions

**DOI:** 10.3390/s25103167

**Published:** 2025-05-17

**Authors:** Yuki Kato, Masayoshi Otaka

**Affiliations:** 1 Department of Intelligent Mechanical Systems Engineering, Kochi University of Technology, 185 Miyanoguchi Tosayamada-cho Kami-city, Kochi 782-8502, Japan; 2 Ono Sokki Co., Ltd., 1-16-1 Hakusan, Midori-ku, Yokohama 226-8507, Japan

**Keywords:** fault diagnosis, compressed sensing, order analysis, operational vibration, sub-Nyquist sampling, data compression, propellers, bearings, gears

## Abstract

In the fields of fault diagnosis and structural health monitoring using sound and vibration, there is increasing interest in data compression techniques based on Compressed Sensing (CS). However, conventional CS approaches that use standard bases such as Fourier or wavelets are unable to achieve sparse representations of operational vibrations in rotating machinery with speed variations, leading to significantly reduced compression performance. To overcome this limitation, this study introduces a CS approach that incorporates order analysis, a technique commonly used in the analysis of rotating machinery. The method constructs an order basis using randomly sampled rotational speed data, enabling sparse observation of operational vibrations through CS. This represents a novel approach for efficiently capturing the essential features of vibration signals under rotational speed variations. The proposed method was validated through numerical experiments. The results showed that for rotational vibrations with speed variations of approximately 10% of the average speed, the compression performance was 20 times higher than that of conventional methods using the Fourier basis. Furthermore, evaluations using simulated vibration signals from eccentric faulty gears, as well as experimental data from defective propellers and bearings with outer ring defects, demonstrated that the proposed method could successfully reconstruct signals even under conditions with substantial speed variation—conditions under which conventional Fourier-based methods fail. Due to its superior compression performance and its ability to handle unknown operational vibrations, the proposed method is highly suitable for applications in fault diagnosis, structural health monitoring, and vibration measurement.

## 1. Introduction

Because failures in rotating machinery can lead to economic losses and accidents, maintaining a good condition through health monitoring is essential. Among several diagnostic methods proposed to achieve this, methods using sound and vibration have been extensively implemented for rotating machinery [1,2], such as bearings [3,4], gearboxes [5,6], and propellers [7,8,9], owing to their robustness and ability to detect faults at early stages. Numerous diagnostic logics have been developed, including classical methods that extract vibration features using fast Fourier transform (FFT), wavelet analysis, and envelope processing [1,4,5,6,8]; methods using machine learning and artificial intelligence [7,9]; and physical model-based methods [2,3,4]. However, these methods involve measuring vibration data based on the sampling theorem, which requires equipment capable of high-speed measurement and a significant amount of data to be transferred and stored. This increases the diagnosis costs and power consumption, thus limiting the actualization of inexpensive diagnosis and wireless sensor-based diagnosis.

A new method for addressing this limitation is based on compressed sensing (CS) theory. CS is a technique for reconstructing the original signal from a small amount of sampled data. This technique can move beyond the constraints of the sampling theorem if it satisfies the conditions that the original signal can be sparsely represented by observing it on a specific basis and that sampling is performed using a random matrix [10]. CS for vibration measurement has been extensively implemented in the field of structural health monitoring using wireless sensor networks [11,12,13,14,15,16,17,18,19,20,21,22,23]. Furthermore, it has been recently used for diagnosing rotating machinery such as bearings [24,25,26,27,28,29,30,31,32,33,34,35,36,37,38,39,40,41], wind turbine equipment [32,35], gears [32,42,43,44,45], propellers [46,47], Diesel engines [48,49], and drilling tools [50]. In these existing studies, the CS of vibrations involved using various basis types, such as Fourier [11,17,18,19,20,46,50], wavelet [11,20,21,22,34,47], complex sinusoids [15], Fourier basis after envelope processing [36], and discrete cosine transform [14,23,33,35,47]. However, none of these can sparsely express the vibration that occurs during actual operations, which is accompanied by speed fluctuations. Another method [12,13,19,25] uses CS combined with sensor data correlation to reconstruct vibrations. However, this method has the disadvantage of leveraging increased amounts of data and measurement costs, as it requires installing several sensors on the same object. Other existing methods have utilized dictionary learning basis [24,40,43,44,45,47,49], machine learning and deep learning [16,32,39], and Bayesian learning [27,48]; however, these methods degrade reconstruction accuracy when conditions are different from those of the training data. Additional methods have been proposed for learning and fault classification without reconstructing signals from compressed data [26,27,28,29,30,31,32,37,38,41], this is summarized in reference [51]; however, performing diagnosis under conditions different from those of the training data for these methods is challenging. In the field of image-based measurement, the authors have previously proposed an order basis capable of sparsely representing operational vibrations [52]. However, the construction of this conventional order basis requires rotational speed data that are fully sampled in accordance with the sampling theorem and, therefore, it does not offer any advantage in terms of data compression.

As described above, existing methods for CS of operational vibrations exhibit poor compression performance, making it difficult to reduce the measurement frequency or compress the data volume effectively. This limitation poses a barrier to the realization of cost-effective diagnostics and the deployment of wireless sensor systems. To overcome this challenge, this study presents, for the first time, a method for constructing an order basis from sub-Nyquist sampled rotational speed data, enabling sparse observation of operational vibrations. The proposed approach achieves significant reductions in both measurement frequency and data volume, thereby enhancing the feasibility of practical and scalable diagnostic systems.

The remainder of this paper is organized as follows: Section 2 describes the analysis method developed in this study, particularly the introduction of order analysis to CS. The experimental setup used to validate the proposed method is explained. The validity of the proposed method was evaluated using numerical calculations, as presented in Section 3. The applicability of the method for failure diagnosis was also evaluated using a signal model that simulates the vibration of a failed gear. Section 4 presents an evaluation of the performance of the proposed method based on experiments using a failed propeller and bearing. Finally, the main findings of the study are summarized in Section 5.

## 2. Materials and Methods

### 2.1. Vibration Measurement Method Using CS with Order-Ratio Basis

The measurement vector *p* ***∈ R****^m^* is obtained using a linear projection of the discrete-time signal *s* ***∈ R****^n^* (*n* > *m*):

(1) p=Φs

where *m* is the number of measurement points, *n* is the number of bases, and ***Φ*** is the measurement matrix. Bernoulli or Gaussian random matrices are typically used in CS. As such, it can be said that ***p*** is the downsampled data of the signal ***s*** through random observations using the measurement matrix ***Φ***. The discrete-time signal ***s*** is represented by the product of the orthonormal basis matrix ***Ψ*** and its coefficient matrix ***q***

(2) p=ΦΨq=Aq

where ***A*** = ***ΦΨ*** is an observation matrix *m × n.* In CS, ***q*** is an unknown variable. In Equation (2), CS reconstructs the original signal from a limited number of sampling points, leading to an underdetermined problem where the number of unknown variables exceeds the number of measurements (*n* ≫ *m*). Consequently, the solution is generally indeterminate and cannot be directly solved. However, if the solution is sparse—meaning that only *s* components of the unknown variable ***q*** have nonzero values while the rest remain zero—then exact reconstruction under the condition *s* ≪ *m* was shown to be possible [53]. Research has also been conducted on the conditions for this to be valid, including rigorous methods for evaluating whether ***A*** satisfies the restricted isometry property (RIP) [53] and methods for assessing the mutual incoherence property (MIP) [54], which represents the degree of incoherence of ***A***. However, both approaches have extremely high computational costs, making it difficult to apply them to problems where ***A*** becomes large, such as high-frequency vibration measurement. Therefore, to establish a method for finding a solution with a realistic amount of calculation, it was shown that when ***Ψ*** is an orthonormal basis, such as a wavelet basis or Fourier basis, using a Gaussian or Bernoulli random matrix for ***Φ*** ensured that ***A*** has RIP under the following conditions [10,55]:

(3) $$m>cs\log\left(n/s\right)$$

Donoho and Tanner showed that when ***A*** is a random matrix, reconstruction is successful, with *c* = 2 being the specific value of *c* [56]. This criterion is often used to reconstruct oscillations using CS, as it is computationally inexpensive [17,46]. Therefore, this value was also used to evaluate the performance of CS in this study. Under the conditions in Equation (3), if a sparse solution satisfying Equation (2) can be found, the signal can be reconstructed. The sparsest solution can be obtained by minimizing the *l*_0_ norm, as shown below

(4) $$\hat{q}={\mathrm{argmin}\left\|q\right\|}_{0}\mathrm{subject}\;\mathrm{to}\;p=Aq$$

However, because Equation (4) is a combinatorial optimization problem, the required calculation is *O* (*_n_*C*_m_*_−1_), which is very large (NP-hard). This issue is often addressed using orthogonal matching pursuit (OMP) [57], which is a kind of fast approximate calculation using greedy algorithms. In this study, OMP was employed. The greedy algorithm approximates the measurement vector ***p*** with only a single component *q_j_*_,_ and this process is repeated *k* times. If the number of components of the original signal is known, the approximation can be repeated as many times as the number of components [58]; however, if the number of components is unknown, setting the termination condition is difficult. When the number of components is unknown, the existing termination conditions can be divided into two main methods as follows:

- The residual in Equation (2) is ***r_e_***, and the criterion for stopping when this energy decreases to a certain value is given by the equation below [59,60].
  ||***r_e_***||_2_ < α (5)
- The criterion for stopping when none of the columns in the observation matrix are strongly correlated with the residual is given by the equation below [61].
  **||*A^H^ r_e_***||_∞_ < *λ_omp_* ||***r_e_***||_2_ (6)

The method using Equation (5) cannot be set up under conditions where the magnitude of noise is unknown because the residual energy α increases or decreases significantly under the influence of noise. Therefore, Equation (5) was not adopted in this study. The method using Equation (6) works under conditions where the magnitude of noise is unknown; therefore, Equation (6) was used in this study. However, because this method may not terminate depending on the noise situation and the value of *λ_omp_*, we set it to stop when the number of components exceeds the number of components in Equation (3). *λ_omp_* exhibits the characteristic that the larger the value, the smaller the number of iterations, *k*; therefore, a sparse solution can be obtained at the cost of a larger error. For *λ_omp_*, because of the high computational cost of MIP-based methods [61], cross-validation is used in this study. *λ_omp_* was calculated in the 0.1–5 range, and the data were divided into five parts. *λ* was gradually increased from 0.1, and the largest coefficients *λ_omp,smax_* were selected for which the mean squared error (MSE) was less than or equal to the MSE + σ (standard deviation) at *λ_omp_* = 0.1.

Another approach to avoiding the NP-hard problem is to minimize the *l_1_* norm. However, *l*_1_-norm minimization is difficult in that the estimation accuracy deteriorates owing to noise; therefore, the following regularization with Lasso is often used:

(7) $$\hat{q}=\mathrm{argmin}\left(\frac{1}{2}{\left\|p-Aq\right\|}_{2}^{2}+\lambda_{l}{\left\|q\right\|}_{1}\right)$$

*λ_l_* is a positive regularization parameter; the larger the value of this parameter, the sparser the solution. In this study, *λ_l_* was obtained using the cross-validation method. In this method, the data were divided into five parts, the MSE was calculated, and the largest coefficient within one standard deviation of the smallest MSE, *λ_l,smax_* was adopted.

**Figure 1:**
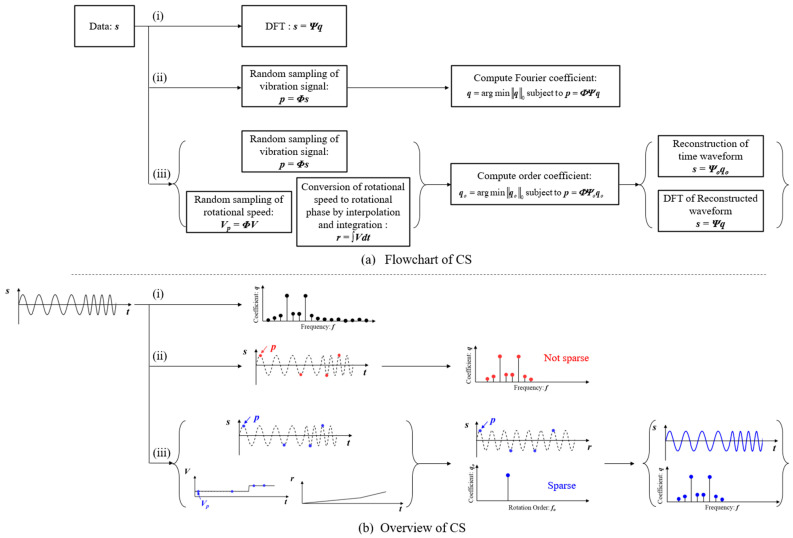
Schematic of CS: (i) DFT, (ii) CS with Fourier basis, (iii) CS with order basis.

Figure 1a presents a flowchart of the proposed vibration measurement method using CS. In frequency spectrum analysis, DFT is typically used, as shown in flow (i) of Figure 1a, which requires full sampling of the signal. To reduce the measurement frequency and compress data volume, CS is applied using a Fourier basis, as illustrated in flow (ii) of Figure 1a. However, as discussed in the introduction, the Fourier basis is not well-suited for sparsely representing operational vibrations that involve speed fluctuations. To address this issue, this study develops an order basis that incorporates rotational speed variation. As shown in flow (iii) of Figure 1a, both the rotational speed and vibration signals are first randomly sampled. The rotational speed data are then interpolated and integrated to obtain the rotational phase. Using this rotational phase information, the order basis is constructed as described below.

(8) $$\left\{\begin{array}{c} \Psi_{o,i}=q_{o,i}\sin\left(f_{o}r\right)\;\;n\leq n/2\\ \Psi_{o,i}=q_{o,i}\cos\left(f_{o}r\right)\;\;n>n/2 \end{array}\right.$$

where *f_o_* is the rotation order ratio, *q*_i_ is the coefficient of the order basis, *n* is the length of the order based on this order basis, and ***r*** is the rotation phase [in radians]. By using this order basis, sparse observation of operational vibrations can be achieved. Based on the coefficients identified using the order basis, as shown at the end of flow (iii) in Figure 1a, the signal is reconstructed and DFT is performed, allowing the frequency spectrum to be obtained.

As a simple example, Figure 1b depicts an overview of the CS in vibration signal when the rotational speed is doubled during the process. In this case, the signal includes frequency modulation; therefore, the spectrum is not sparse, as shown in flow (i) of Figure 1b, and CS using the Fourier basis is difficult. Therefore, observation using the order basis is carried out. When the change in the vibration signal is plotted against the rotation phase, the waveform is almost sinusoidal, as shown in the center of flow (iii) of Figure 1b. CS with order basis yields the sparse spectrum shown on the center of flow (iii) of Figure 1b. By reconstructing this spectrum using Equation (2) and reconstructing the time information, the operational vibration can be recovered, as shown in the right of flow (iii) of Figure 1b. Table 1 presents the basis conditions used in this study. In the Fourier basis, the Nyquist frequency was used as the upper limit. For the order basis, the upper limit was set to the order corresponding to the Nyquist frequency.

### 2.2. Verification Equipment for Propeller Failure Diagnosis

**Figure 2:**
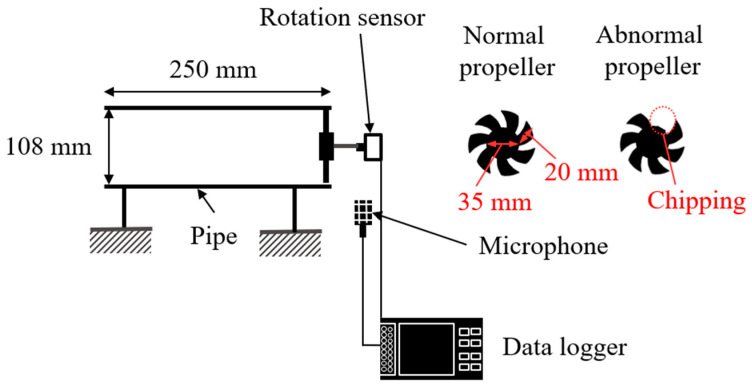
Experimental setup for the fault diagnosis of a propeller.

An experimental apparatus was designed to verify the fault diagnosis method for rotating machinery using CS, as shown in Figure 2. The apparatus was used in an existing study [46] in which sparse observation was achieved on the Fourier basis because the rotational speed was controlled at a constant level. However, in this experiment, a constant current was supplied to the propeller drive motor without controlling the rotational speed; therefore, the rotational speed fluctuates unsteadily under the influence of fluid forces. Variations in the fluid sound caused by an abnormal propeller were measured using a microphone (PCB Piezotronics, Depew, NY, USA, 130A24) installed at the pipe outlet. This microphone is a free-field type capable of measurements in the 20–16,000 Hz range with an uncertainty of ±3 dB. A data logger (Ono Sokki, Yokohama, Japan, DR-7100) was used to record the data at a sampling rate of *f_s_* = 1280 Hz. The rotation pulse (once per revolution) was measured using a rotation sensor (Sensatec, Kyoto, Japan, SEELV-24W36G) and converted to rotational speed. Although capturing the rotational speed directly using a rotational speedometer is a more efficient process when operating as a failure diagnosis device, the rotational pulses were measured for verification. Because the measurement time was set to *T_s_* = 30 s, the measurement data length for full sampling was *n* = 38,400. As shown in Figure 2, failure was simulated by cutting one of the seven blades. The propeller rotation frequency was set to *f_p_* ≈ 26 Hz; therefore, the propeller passing frequency was *f_pp_ = f_p_* × 7 ≈ 182 Hz. Because the rotation frequency and the propeller pass frequency are both lower than the Nyquist frequency, *f_s_*/2 = 640 Hz, one would expect to be able to determine the effect of the fault from the frequency spectrum.

### 2.3. Verification Equipment for Bearing Failure Diagnosis

**Figure 3:**
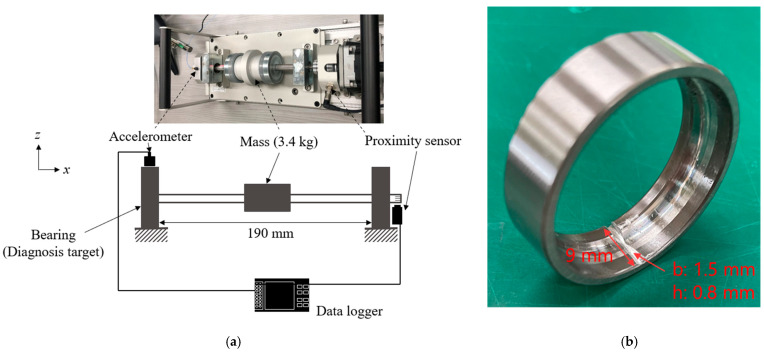
Bearing test equipment: (**a**) schematic and photograph, (**b**) bearing with a defective outer ring.

An experiment was conducted to evaluate the reconstruction performance of the proposed method for bearing failure using a bearing rotating device, as shown in Figure 3a. The rotational speed was set to 2400 min^−1^; therefore, the rotational frequency was *f_r_* = 40 Hz. A normally operating bearing and a bearing with outer ring failure, as shown in Figure 3b, were constructed, and the driving vibration of each was measured. The size of the outer ring failure was 1.5 mm wide and 0.8 mm deep; therefore, we estimated that the failure would cause a noticeable change in the vibration signal. Angular contact ball bearings (NSK, Tokyo, Japan, 7002ADB) were used; the size of each part is listed in Table 2. The frequency of the generated impact vibration (ball pass frequency outer: BPFO) is expressed as follows [1]:

(9) $$f_{out}=\frac{1}{2}f_{r}\left(1-\frac{d}{D}\cos\theta_{c}\right)$$

An accelerometer (Ono Sokki, Japan, NP-3211) was installed on top of the bearing to measure *z*-direction vibrations. A data logger (Ono Sokki, Japan, O-Solution DS-5000) was used for the data measurements with a sampling frequency of *f_s_* = 5120 Hz. The measurement time was set to *T_s_* = 10 s such that the number of measurement data points for full sampling was *n* = 51,200. The uncertainty of the accelerometer was 5% in the range of 1–10 kHz. A proximity sensor (Omuron, Kyoto City, Japan, E2E-X1R5E1-M3) was used to detect the number of rotational pulses, which was converted to rotational speed using the external pulse input of the data logger and the internal calculation function.

## 3. Numerical Experiment

### 3.1. Verification of the Principle of CS with Order Basis

We generated a simulated vibration waveform and performed random sampling and reconstruction of the signal to evaluate the difference between the CS performance of the Fourier basis and order basis for rotational vibration. Given a system oscillating at the 2nd, 5.12, and 10th orders of rotation, the oscillating signal can be expressed as follows:

(10) $$s_{nu}=\sin\left(4\pi i_{o}\int_{0}^{t}f_{r}\left(t\right)dt+1\right)+\sin\left(10.24\pi i_{o}\int_{0}^{t}f_{r}\left(t\right)dt+1\right)+\sin\left(20\pi i_{o}\int_{0}^{t}f_{r}\left(t\right)dt+1\right)$$

The initial phase and amplitude are set to 1. The rotational frequency is set using the following equation:

(11) $$f_{r}\left(t\right)=50+5\left(t-1\right)/2$$

*T_s_* = 2 was used in the calculations so that the peak-to-peak range of the rotational frequency variation was 10% of the average. Sampling frequency: *f_s_* = 1280 Hz, resulting in a total signal length of *n* = 2560.

**Figure 4:**
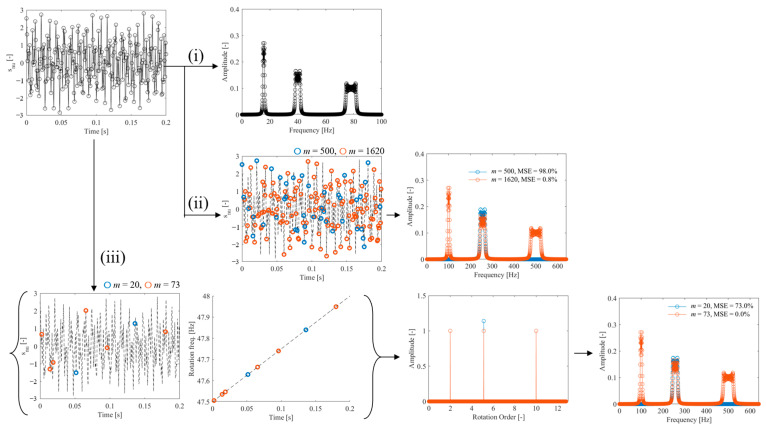
CS of operational vibration under speed variation conditions: (i) DFT, (ii) CS with Fourier basis, (iii) CS with order basis (calculated using Lasso {Equation (7)}).

The top-left of Figure 4 shows the vibration waveform generated using Equations (10) and (11). This signal was processed according to the flowchart in Figure 1, following the procedures outlined in flows (i), (ii), and (iii). In flow (i) of Figure 4, the frequency spectrum was calculated using the DFT with a rectangular window. Peaks appear at 100 Hz, 256 Hz, and 500 Hz, corresponding to the 2nd-, 5.12-, and 10th-order components of rotation, respectively. According to Equation (10), the bandwidth of each peak increases with order due to greater sensitivity to rotational speed variation. By counting spectral components with amplitudes exceeding 0.01, the number of sine and cosine terms was found to be 490. Therefore, based on Equation (3), at least *m* = 1620 measurement points are required for CS with Fourier basis. Flow (ii) of Figure 4 shows the results of random sampling and frequency spectrum estimation using CS with a Fourier basis. The number of measurement points was set to *m* = 500 and *m* = 1620, and the MSE was evaluated using the spectrum from flow (i) as the reference. The results indicate that MSE becomes 0.8% when the number of points meets or exceeds the theoretical requirement (*m* = 1620). However, the corresponding compression ratio is 0.63, which is relatively high, indicating insufficient data reduction. In contrast, the left side of flow (iii) in Figure 4 shows the random sampling of vibration and rotational speed signals with *m* = 20 and *m* = 73 points. Although the number of variables increases compared to flow (ii), the total number of sampling points is significantly reduced. The right side of flow (iii) shows the order spectrum obtained using CS with an order basis. The spectrum clearly exhibits a sparse structure, and in the case of *m* = 73, the three rotational order components specified in Equation (10) are accurately identified. According to Equation (3), the minimum number of measurement points required to identify these three rotational vibration components is *m* = 73, confirming that the identification was performed accurately at the theoretical limit. Next, following flow (iii) in Figure 1, the vibration signal was reconstructed using this order spectrum, and the frequency spectrum was calculated via DFT. As expected, the frequency spectrum achieved an MSE of 0.0% at *m* = 73. Furthermore, the compression ratio was 0.029, which is less than one-twentieth of that obtained using the Fourier basis, demonstrating the high efficiency and effectiveness of the proposed method.

**Figure 5:**
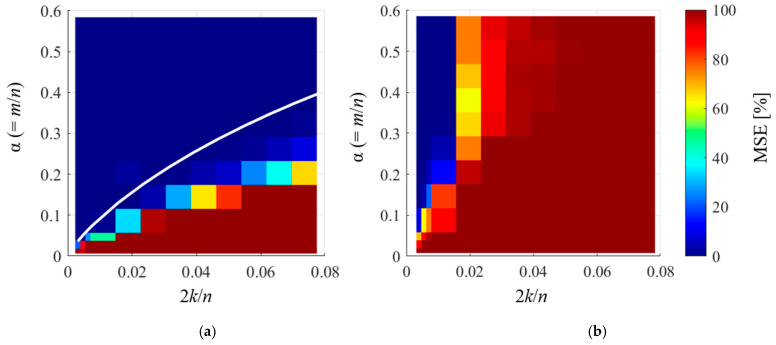
Phase transition diagram in CS of rotational vibration (calculated using the proposed method {OMP, Equation (4)}): (**a**) order basis, (**b**) Fourier basis.

According to Equation (8), the order basis has the same properties as the Fourier basis, except that the timestamp changes with the rotation speed; therefore, it is expected to satisfy the RIP and the Fourier basis. However, because a new *l*_0_ minimization algorithm was used in this study, we conducted numerical experiments to evaluate whether CS with the order basis is feasible within Equation (3). The following signals were used in the numerical experiments:

(12) $$s_{nu}=\sum_{j=1}^{k}A_{j}\sin\left(2\pi i_{o,j}\int_{0}^{t}f_{r}\left(t\right)dt+\phi_{j}\right),$$

where *φ_j_* is the phase and *A_j_* is the amplitude, which are variables that take random values in the interval 1–10. Through numerical experiments using Equation (12), we evaluated the reconstruction error of CS under different measurement points and components and created the phase transition diagram shown in Figure 5. The vertical axis shows the number of measurement points (*m*), and the horizontal axis shows the double-order component (2*k*). The contours show the mean MSE values for ten numerical experiments with different random matrices for each condition. The results in Figure 5a show that the MSE is almost zero in the region where the number of vibration components is small and the number of measurement points is large, indicating that the reconstruction with order basis was successful under a wide range of conditions. Furthermore, the theoretical line when *s* = 2*k* in Equation (3) is represented by a white line, which indicates that the accurate reconstruction was conducted under all conditions within the range of Equation (3). By contrast, the Fourier basis results in Figure 5b show that the reconstruction performance is significantly worse, with a wider range of failed reconstructed regions. This is because the Fourier coefficient increases by approximately 80 times for each additional rotational component, as shown in flow (ii)–(iii) of Figure 4, which increases the number of points required for reconstruction by approximately 20 times.

From the above results, it can be deduced that the use of CS with the order basis can accurately recover the vibration within the range of Equation (3). In addition, the proposed order basis yields a significantly better compression performance than the Fourier basis used in existing studies [11,17,18,19,20,46,50] for measuring rotational vibrations via CS.

Next, we evaluated the effect of the interpolation method on rotational speed. We observe that for a linear change in the rotational speed, as given in Equation (11), the true speed can be obtained using linear interpolation. However, in rotating machinery, the rotational speed often fluctuates periodically and is unstable owing to the effects of engine combustion and fluid forces on the propeller. If the periodic fluctuations are stable, the rotational speed can be determined by CS. However, if the fluctuations are unstable and have a short period, as is the case in actual equipment, interpolation using a function is expected to be appropriate. Therefore, we evaluated the effects of linear and spline interpolations on the reconstruction performance. Equation (10), which has three rotational orders, was used as the vibration signal. The variations in the rotational frequency are expressed as given in the following equation:

(13) $$f_{r}\left(t\right)=50+5\left(t-1\right)/2+\sin\left(2\pi t\right)$$

**Figure 6:**
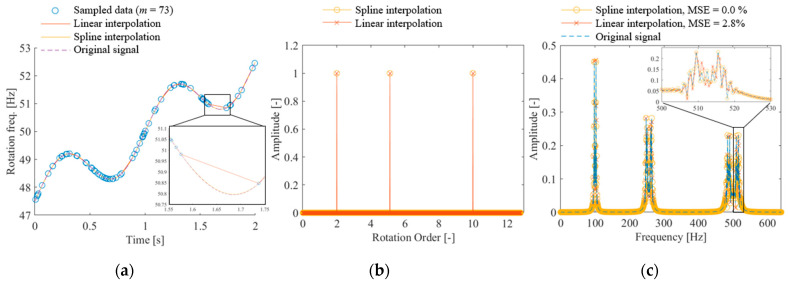
CS of rotational oscillations with low periodic speed fluctuations (calculated using the proposed method {OMP, Equation (4)}): (**a**) time variation of rotation frequency, (**b**) CS with order basis, (**c**) frequency spectrum of reconstructed signal.

Figure 6a shows the time-series variations in the rotational frequency defined in the above equation. The dotted line shows the original full-sample data, and the circle plot shows the randomly sampled data. The solid line shows the results of completion processing using spline or linear interpolation based on the randomly sampled data. Although the original waveform is approximately reconstructed, as shown in the enlarged figure, linear interpolation fails when the spacing between the data points is large. Figure 6b shows the CS results with the order basis using the rotational phase integrated using each interpolation method. Both interpolation methods accurately identify the vibration components. Figure 6c shows the frequency spectrum calculated using Fourier transform after reconstructing the vibration. From the figure, it can be observed that the result from the spline interpolation agrees well with the original signal. However, linear interpolation yields minor differences, indicating that the signal is not completely reconstructed. The 2.8% MSE can be attributed to the rotation frequency completion error shown in Figure 6a. The figure shows that no difference exists between the completion methods in identifying the vibration components; therefore, the effect on failure diagnosis is negligible. However, spline completion should be used to accurately reconstruct the original vibration.

**Figure 7:**
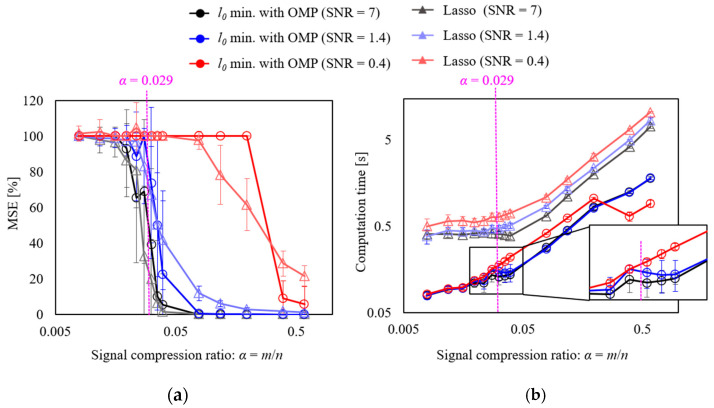
Evaluation of the MSE and computation time for CS with order basis: (**a**) MSE, (**b**) computation time.

Next, the vibration signal in the following equation was generated by adding noise to Equation (10), and the effect of noise on reconstruction accuracy was evaluated.

(14) $$\begin{array}{c} s_{nu}=\sin\left(4\pi i_{o}\int_{0}^{t}f_{r}\left(t\right)dt+1\right)+\sin\left(10.24\pi i_{o}\int_{0}^{t}f_{r}\left(t\right)dt+1\right)\\ +\sin\left(20\pi i_{o}\int_{0}^{t}f_{r}\left(t\right)dt+1\right)+n\left(\sigma ,t\right) \end{array}$$

where *n* is the Gaussian noise, and the standard deviations σ are set to 0.1, 0.5, and 1.0. The signal was reconstructed using the proposed method (OMP, Equation (4)) and Lasso (Equation (7)), and the results of the MSE evaluation are shown in Figure 7a. The error bars represent the standard deviation of the MSE after generating ten random matrices with different random matrices and signal reconstruction. First, the reconstruction results obtained using the proposed method, which are shown in the circle plot in Figure 7a, are discussed. From the results, it can be observed for a signal-to-noise ratio (SNR) of 7 that the MSE converges to 0 and the variations become small at a theoretical value of *α* = 0.029 (*m =* 73) or more, as given in Equation (7). Therefore, highly accurate measurements are possible. When the SNR is 1.4 and the noise is large, a signal compression ratio approximately twice that of the theoretical value is required. When the SNR is 0.4 and the noise is very high, the MSE does not converge unless the signal compression ratio is more than ten times that of the theoretical value. Therefore, the proposed method is effective when the SNR is greater than 1. Comparing the methods, at SNRs of 1.4 and 0.4, the MSE of the proposed method converges to 0 with a lower signal compression ratio than that of Lasso. This is because in the Lasso method (Equation (7)) when the noise is large and the value of λ is large, the amplitude is underestimated owing to the effect of the *l_1_* regularization term. Therefore, increasing the number of data points and reducing the effect of the *l_1_* regularization term are necessary to accurately estimate the value using Lasso under conditions in which the noise is large. In the proposed method, underestimation of the amplitude cannot occur as it is a minimization of *l*_0_.

Next, the computation time was calculated to determine the computational cost of the reconstruction method. Figure 7b shows the relationship between the signal compression ratio: *α* and computation time. A laptop computer (memory: 32 GB, CPU: Intel Core Ultra 9 185H) was used for the calculations. The result shows that the computation time of the proposed method is significantly shorter than that of Lasso in all SNR conditions. This is particularly true when the SNR is small. In Lasso, more calculation time is required as the noise increases, whereas the proposed method is an algorithm that adds support vectors using the greedy method; therefore, the effect of noise is minimal and the computation time is almost the same for noise below an SNR of 1.4. Based on the computation time of the proposed method at an SNR of 1.4–7, an inflection point can be observed around α = 0.029. In addition, a region exists, in which the computation time decreases even when the signal compression rate increases. This is because values close to the true solution can be identified when α = 0.029 or more; therefore, the calculation is terminated when *λ* is small. When the SNR is 0.4, the true solution can be identified when α is approximately 0.4; therefore, the inflection point is in this region. In the case of the method using Lasso, the computation time exceeded the data duration of 2 s when α was set to 0.2 or higher. Therefore, real-time processing under these conditions is likely to be difficult. On the other hand, the method using OMP maintained the computation time within 2 s for all tested conditions, suggesting its potential for real-time processing. However, since this study utilized a high-performance laptop computer, there is a possibility that a typical data logger may lack sufficient computational power. Therefore, in order to achieve real-time fault diagnosis in practical environments, it would be desirable to transfer the randomly sampled data to a processing PC or a cloud server for signal reconstruction. This approach would significantly reduce the computational load and power consumption on the sensing device, making real-time fault diagnosis feasible.

The above results indicate that the proposed method has a short computation time and requires a small number of measurement points under conditions where noise is present. Therefore, all vibration restorations in the following CS sections were performed using OMP.

### 3.2. Evaluation of CS with Order Basis for Gear Failure

In this section, we evaluate a case in which gear failure diagnosis is performed using the proposed method. Although modeling all gear meshing vibrations is difficult owing to their complexity, their behavior in the low-speed range can be represented using a simple signal model [62]. Therefore, in this study, a simplified model was used to numerically verify the proposed method. First, the meshing vibration caused by a gear rotating at low speed can be expressed as given in the following equation:

(15) $$s_{gear}=A\sin\left(2\pi f_{m}t\right)$$

where *f_m_* (=*f_r_ Z*) is the tooth engagement frequency, which can be expressed by multiplying the rotation frequency *f_r_* by the number of teeth, *Z.* In this study, Z is set to 40 and *f_r_* is set using the following equation:

(16) $$f_{r}\left(t\right)=10+(t-1)/2\;\;\;\;\;:\;\mathrm{Linear}\;\mathrm{increase}$$

(17) $$f_{r}\left(t\right)=10+(t-1)/2+\sin\left(2\pi t\right)\;\;\;:\mathrm{Linear}\;\mathrm{increase}\;+\;\mathrm{FM}\;\mathrm{modulation}$$

**Figure 8:**
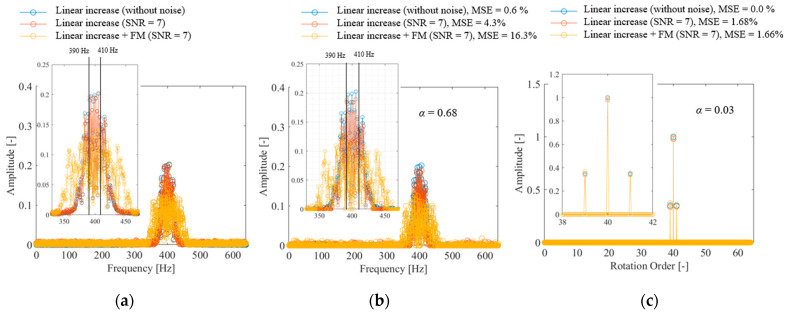
Spectrum of gear vibration: (**a**) DFT, (**b**) CS with Fourier basis, (**c**) CS with order basis.

Several types of gear failures exist, such as eccentricity, misalignment, meshing error, pitch error, and tooth chipping. This study focused on eccentricity as a case study. When eccentricity occurs in the bearing, tooth meshing vibration causes amplitude modulation with rotational frequency, as given in the following equation:

(18) $$s_{gear}=\{1+A_{h}\sin\left(2\pi f_{r}t\right)\}A\sin\left(2\pi f_{m}t\right)+n(\sigma ,t)$$

where *A_h_* is the magnitude of the amplitude modulation, which was set to 0.7 in this case. *n* is the Gaussian noise, and the standard deviation σ was set to 0 and 0.1. Figure 8a shows the frequency spectrum of the meshing vibration of a normal gear generated using Equation (18). The blue plot for the noiseless condition shows a peak near 400 Hz, corresponding to the gear engagement frequency. However, the bandwidth of the spectrum is large and not sparse owing to the effect of fluctuations in the rotation frequency. In addition, a sideband wave due to amplitude modulation should exist at *f_m_* ± *f_r_* Hz; however, it is buried under the effect of rotation frequency fluctuation and cannot be identified. When noise is added or when the rotational frequency is modulated owing to gear fault, the bandwidth is even wider and the spectrum is not sparse. Figure 8b shows the result of CS using the Fourier basis with a signal compression ratio of α = 0.029. The spectrum observed is not sparse; hence, spectrum identification fails. Figure 8c shows the results of CS using the order basis with a signal compression ratio of α = 0.029. The order spectrum is sparse, and the 40th-order component of the rotation and its sidebands can be clearly identified. When the signal was reconstructed, the MSE was less than 2% under all conditions, indicating that highly accurate measurements were obtained. As shown in Figure 8c, the sidebands, which could not be detected using DFT, can be clearly detected using the proposed method; therefore, it is expected that the degree of failure can be diagnosed by monitoring the amplitude of the sidebands using the proposed method. Although similar effects were reported in previous studies [63,64,65], it is noteworthy that spectral sharpening can be achieved with a small measurement point.

**Figure 9:**
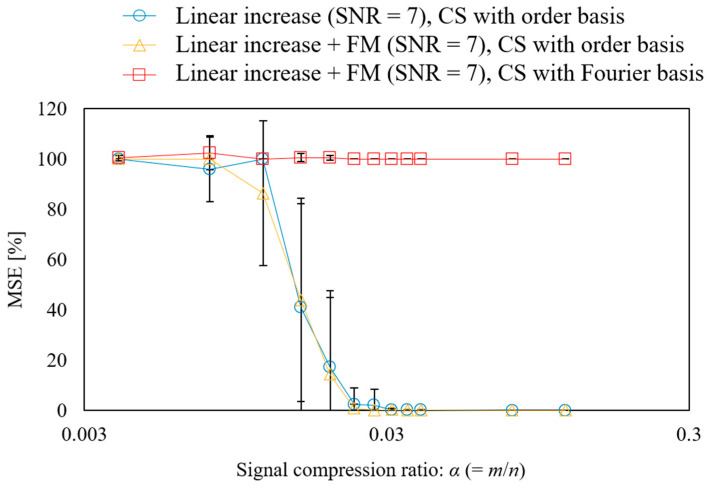
Effect of signal compression ratio on the CS of gear vibration.

Next, the relationship between the signal compression ratio and MSE was evaluated, as shown in Figure 9. The plot shows the arithmetic mean of the results of ten reconstructions using different random matrices, and the error bars show the standard deviation. The result shows that in CS with order basis, the MSE becomes almost zero when a signal compression ratio of α = 0.029 or higher is used, which is a theoretical value. In addition, the error bars are almost zero. In terms of the form of the rotational speed fluctuation, measuring linearly rising and low-period fluctuations in the same manner is possible. In the case of CS using the Fourier basis, the error remained large even when the signal compression ratio increased. This is because the spectrum is not sparse in the Fourier basis owing to the effect of rotational speed fluctuations, as shown in Figure 8a.

The above results show that the proposed order basis is more effective for gear vibrations compared with the Fourier basis used in previous studies [11,17,18,19,20,46,50]. Because order-ratio analysis can be performed simultaneously, the proposed method can clearly detect sidebands that were unclear when FFT was used. In addition, it can achieve extremely sensitive fault diagnosis.

## 4. Experimental Results

### 4.1. Evaluation of CS with Order Basis for Propeller Fault Diagnosis

**Figure 10:**
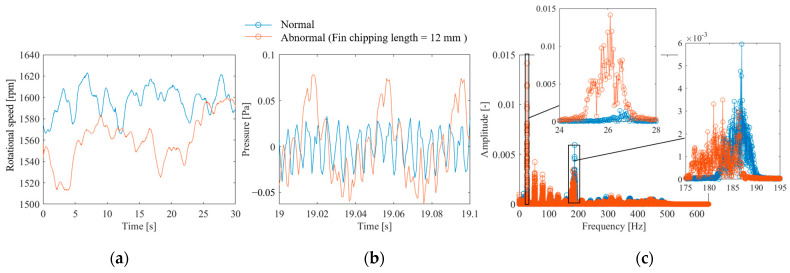
Measurement results of propeller-driven sound: (**a**) rotational speed waveform, (**b**) sound pressure waveform, (**c**) DFT results of sound pressure.

In this study, a propeller drive test was conducted using the apparatus shown in Figure 2 to evaluate the applicability of the proposed method for propeller failure diagnosis. Figure 10a shows the measured rotational speed. The rotational speed fluctuates unstably under both normal and anomalous conditions. Such rotational speed fluctuations are possibly due to the fluid force of the fan and generally occur except when special measures are implemented, such as driving with a stepping motor, controlling the rotational speed, or adding an inertial mass. The time-averaged rotational frequencies in the experiments for the normal and abnormal propellers were *f_r_* = 26.6 and 26.0 Hz, respectively. The time-averaged propeller passing frequencies were thus *f_p_* = 186.2 and 183.0 Hz, respectively. The waveform of the sound pressure fluctuation is shown in Figure 10b. The figure shows that the normal propeller-driven sound fluctuates at *f_p_* = 186.2 Hz, which is the propeller passing frequency. However, the sound driven by the anomalous propellers has a low-frequency rotation component in addition to the propeller passing frequency. This is expected because the presence of a missing propeller causes an imbalance in the flow generated by the propeller, which synchronizes with the rotational frequency. The Fourier transform of the pressure waveform is shown in Figure 10c. The amplitude of the propeller passing frequency is large under normal conditions, whereas the rotation frequency and its order component are large under anomalous conditions. In addition, the bandwidth of each peak is large owing to the effect of rotation frequency fluctuations, indicating that the Fourier basis is not sparse.

**Figure 11:**
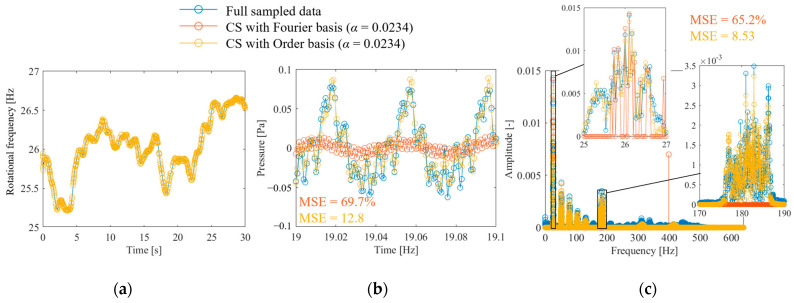
CS of propeller-driven sound: (**a**) rotational speed waveform, (**b**) sound pressure waveform; (**c**) DFT results of sound pressure.

Figure 11 shows the results of CS of anomalous propeller sound using the proposed method. Figure 11a shows the full-sampled and random-sampled data for the rotation frequency. Because of the long period of fluctuation, even random sampling with a low signal compression ratio of *α* = 0.023 is sufficient to track the fluctuations. Figure 11b shows the results of reconstructing the vibration signal from the rotational speed using the flow shown in Figure 1. The results of CS with order basis shown in the yellow plot indicate that both the fine fluctuations at the propeller passing frequency and the large fluctuations at the rotational frequency are reproduced. However, CS with the Fourier basis shown in the orange plot failed to reconstruct the propeller passing frequency component and underestimated the rotation frequency variations. Figure 11c shows the results of the frequency spectrum comparison. The figure shows that CS with an order basis can measure the first-order rotation, its harmonic components, and the propeller passing frequency component. By contrast, CS with the Fourier basis only measures a portion of the rotational frequency component and has a very large MSE of 65.2%. In addition, misestimation occurred, as indicated by the peak near 400 Hz.

**Figure 12:**
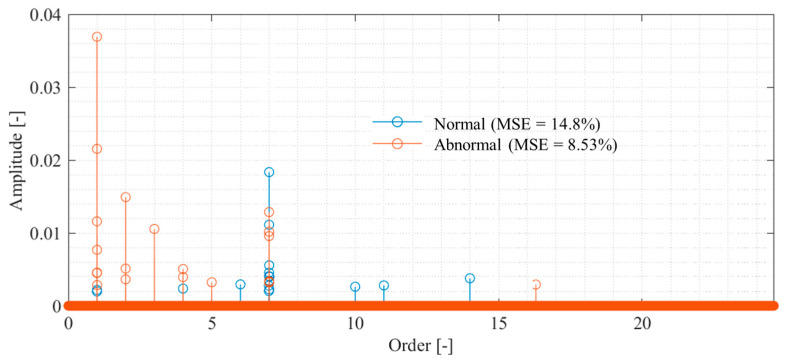
Order spectrum of propeller-driven sound identified via CS with order basis, α = 0.023.

The order spectrum was identified to perform propeller failure diagnosis using the measurement results from the proposed method, and the results are shown in Figure 12. Because the order components can be identified sparsely, the effect of the presence or absence of failure can be easily evaluated. Figure 12 shows that both spectra have peaks in the first- and seventh-order components. The first-order rotational component is large for an abnormal propeller, and the seventh-order rotational component is large for a normal propeller. Therefore, monitoring the increase in the rotation frequency component will enable fault diagnosis. Even if the rotational frequency component increases in normal propeller conditions owing to eccentricity or other factors, malfunctions can be diagnosed by monitoring the decrease in the propeller passing frequency component.

**Figure 13:**
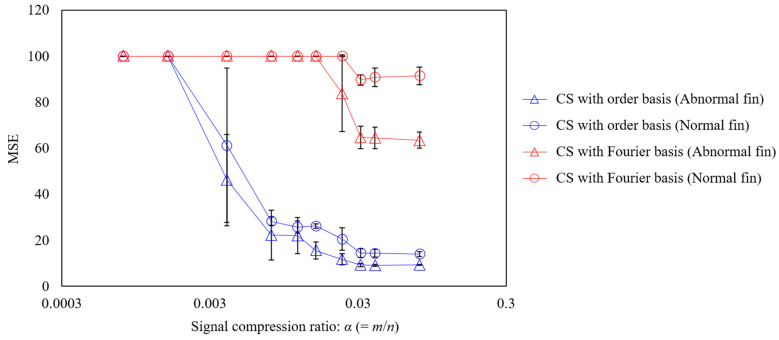
Relationship between the signal compression ratio and MSE for the CS of propeller-driven sound.

The relationship between the signal compression ratio and the measurement error is shown in Figure 13. The plot shows the arithmetic mean of the results of ten reconstructions using different random matrices, and the error bars show the standard deviation. The results shown in the blue circle plots, which reconstruct the vibration under normal propeller conditions using CS with an order basis, show that the MSE and standard deviation converge to zero at a signal compression ratio of approximately 0.029 or higher, achieving good measurement. The same trend is seen in the results for the abnormal propeller conditions shown in the triangular plot; however, the MSE is even smaller than that for the normal propeller conditions. This is assumed to be due to the SNR being smaller because the vibration component of the abnormal propeller is larger. Conversely, the results of CS using a Fourier basis are shown in the red triangle plots of Figure 13, in which even setting the signal compression ratio to 0.029 or higher leads to the MSE exceeding 60%. In the results for the anomalous propeller shown in the red circle plots, the MSE exceeds 90%. This shows that the order basis is more suitable for CS of propeller-driven sound than the Fourier basis used in previous studies [11,17,18,19,20,46,50].

**Figure 14:**
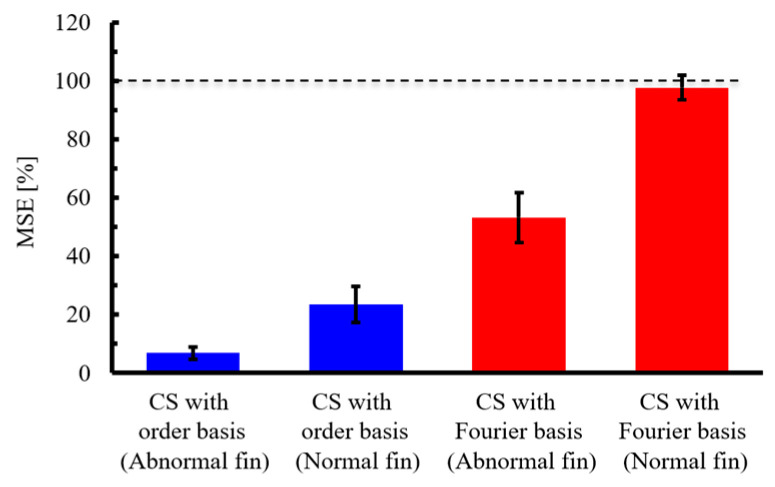
Mean and standard deviation of the MSE for different experimental datasets. CS was performed under the condition of a signal compression ratio: α = 0.0315. The dashed line indicates 100% MSE.

Next, five different experiments were conducted, and for each experiment, ten different random sampling patterns were applied to calculate the average MSE. Subsequently, the mean and standard deviation of these MSE values across the five experiments were evaluated, as shown in Figure 14. From the figure, it can be observed that CS with the order basis yields a lower MSE than CS with the Fourier basis, for both the abnormal and normal propeller cases. In each case, the difference in MSE between the two bases exceeds the standard deviation, indicating that the superior compression performance of CS with the order basis is statistically significant.

### 4.2. Evaluation of CS with Order Basis for Bearing Fault Diagnosis

**Figure 15:**
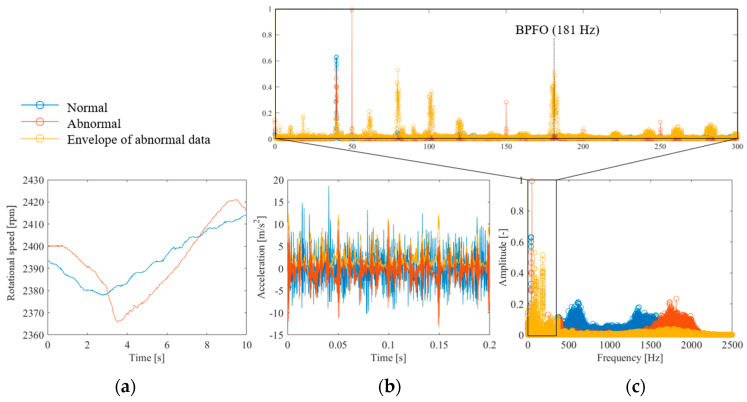
Measured values of bearing vibration: (**a**) rotational speed waveform, (**b**) accelerometer waveform, (**c**) frequency spectrum of acceleration.

We conducted experiments using the apparatus shown in Figure 3 to evaluate the applicability of the proposed method for bearing fault diagnosis. Figure 15a shows the measured shaft rotational speed. The rotational speed fluctuates significantly in both normal and anomalous conditions. Figure 15b shows the time waveform of the accelerometer installed above the bearing. The normal and anomalous bearings both exhibited high-frequency vibrations. In addition, a damped shock waveform was observed under anomalous conditions. This is the result of the impact generated by the rolling element passing through the outer ring defect being amplified by the natural vibration of the bearing [1], which is a common phenomenon. Figure 15c shows the Fourier transform results for these waveforms. In anomalous bearings, many peaks can be observed in the high-frequency range of 1500–2000 Hz. Because the onset interval of the shock amplified by this natural vibration corresponds to the BPFO [1], information on the onset interval was extracted via envelope processing. The yellow plot in Figure 15b shows the results of envelope processing using the Hilbert transform. The figure shows that the envelope of the shock waveform was extracted. Figure 15c shows the results of the Fourier transform. A peak consistent with BPFO was observed, which is similar to the result reported in a previous study [1]. The above results lead to the conclusion that fault diagnosis is possible if a frequency spectrum containing frequency components in the range of 0–2500 Hz can be obtained.

**Figure 16:**
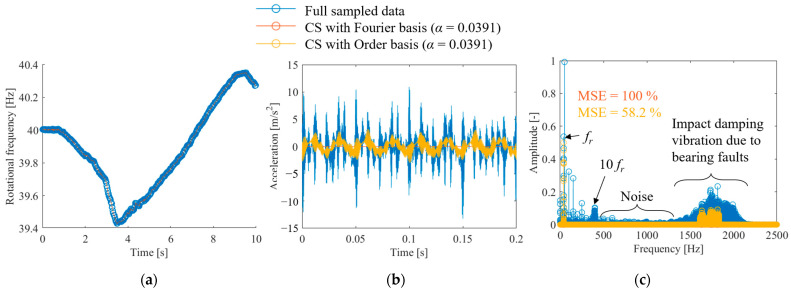
CS of the vibration waveform during bearing anomaly: (**a**) rotational speed waveform, (**b**) accelerometer waveform, (**c**) frequency spectrum of acceleration.

Figure 16 shows the results of CS anomalous bearing vibrations using the proposed method. Figure 16a shows the full-sampled and random-sampled data for the rotational frequency. The figure shows that owing to the long fluctuation period, random sampling with α = 0.0391 is sufficient to track the fluctuations. Figure 16b shows the results of reconstructing the vibration signal from random sampling using the flow shown in Figure 1. The results of CS with the order basis show that some of the shock-damped waveforms caused by anomalies can be reconstructed. By contrast, CS using the Fourier basis yielded a value of zero, indicating that the reconstruction failed. The frequency spectrum is shown in Figure 16c. CS using the order basis can reconstruct first-order rotation and part of the 1500–2000-Hz component. However, CS using the Fourier basis failed to identify any component.

**Figure 17:**
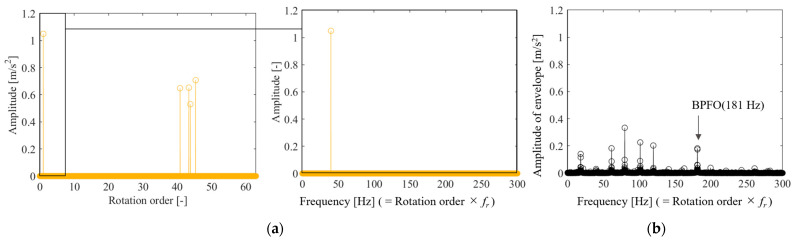
CS of the vibration waveform during bearing anomaly: (**a**) order spectrum; (**b**) frequency spectrum of the envelope.

Figure 17a shows the results of spectral identification with the order basis. The figure shows that first-order rotation and 40–50th-order rotation components were observed. The envelope processing results of the reconstructed waveform in the time domain and its Fourier transform are shown in Figure 17b. The figure shows that envelope processing of the CS results using the order basis can detect outer ring failures. However, as shown in Figure 16c, only a portion of the 1500–2000 Hz range was reconstructed, and the MSE was also large. The reason for the large error is possibly because the impact waveform contains many components in the 1500–2000 Hz band; therefore, the number of variables to be identified increases. In addition, because the impact vibration is a damped vibration waveform, the peak bandwidth is large, resulting in a spectrum that is not sparse, which can increase reconstruction difficulty. Solving this problem requires a basis that can sparsely represent shock waveforms, which we expect to achieve by combining it with a method that decomposes shock waveforms, such as dynamic mode decomposition [66,67].

**Figure 18:**
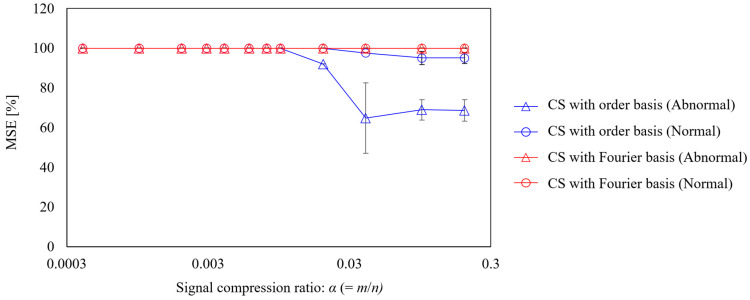
Signal compression ratio against MSE for the CS of bearing vibration.

Finally, the effect of the signal compression ratio on the reconstruction error was evaluated, as shown in Figure 18. The plot shows the arithmetic mean of the results of ten reconstructions using different random matrices, and the error bars show the standard deviation. First, for CS using the Fourier basis, the MSE was 100%, even when the signal compression ratio was greater than 0.2. This is because the vibration in the Fourier domain is not sparse, as shown in Figure 15c and Figure 16c. By contrast, when the vibration of the anomalous bearing was reconstructed using the proposed method, the MSE converged to approximately 60% under conditions where α is 0.1 or greater, and the variation also became smaller. However, the MSE was very large compared with the results for the gear vibration shown in Figure 9 and the propeller-driven sound shown in Figure 13. As shown in Figure 16c, this is owing to the inclusion of noise components and impact vibrations caused by defects in the outer ring, in addition to the rotational frequency vibration. Although these shock waveforms are excited synchronously with the rotation frequency, as mentioned above, they do not form a sparse spectrum, which possibly deteriorates the CS performance. The results of reconstructing the vibration of the normal bearing via CS using the order basis show that the MSE is approximately 90% even when α is greater than 0.1, which is significantly worse than the performance against a failed bearing. As shown in Figure 15c, this is expected owing to the absence of a shock vibration component synchronized with the rotational frequency, in addition to a larger noise component.

**Figure 19:**
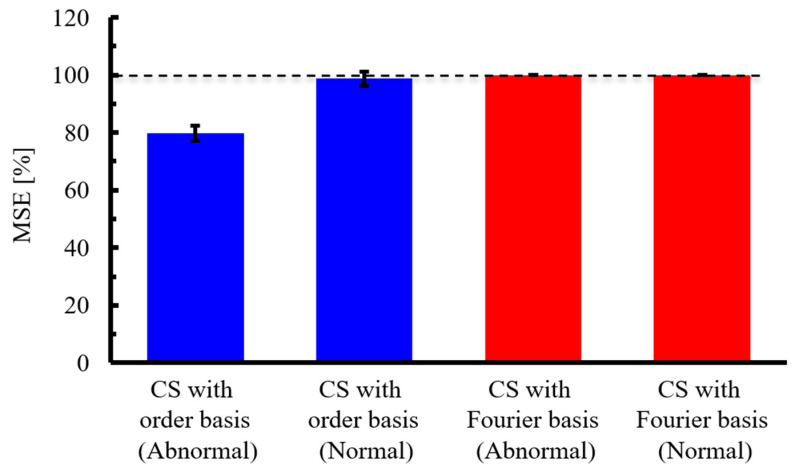
Mean and standard deviation of the MSE for different experimental datasets. CS was performed under the condition of a signal compression ratio: α = 0.1. The dashed line indicates 100% MSE.

Next, five different experiments were conducted, and for each experiment, ten different random sampling patterns were applied to calculate the average MSE. Subsequently, the mean and standard deviation of these MSE values across the five experiments were evaluated, as shown in Figure 19. From the figure, it can be observed that for the abnormal bearing data, CS with an order basis yields a lower MSE than CS with a Fourier basis. Moreover, the difference in MSE between the two methods exceeds the respective standard deviations, indicating that the order basis offers statistically superior reconstruction performance. In contrast, for the normal bearing data, the MSE remains high—above 95%—even with CS with order basis, likely due to the degradation of SNR, as mentioned earlier. In this case, the difference in MSE between the Fourier and order bases is smaller than the standard deviation, and thus the statistical superiority of the order basis cannot be confirmed. However, since the MSE of the order basis is smaller than CS with Fourier, there is potential for improved signal reconstruction through noise reduction strategies.

## 5. Conclusions

In this study, a CS method based on a rotational speed-based order was developed to compress data and reduce the sampling frequency in the fault diagnosis of gears, propellers, and bearings under operating conditions with rotational speed fluctuations. Numerical experiments were conducted to verify the performance of the proposed method and its applicability for gear failure diagnosis. The applicability of the proposed method for propeller and bearing failure diagnosis was also evaluated through experiments. The following conclusions are drawn based on the study findings:

- Numerical experimental results showed that for the CS of rotational vibrations with a velocity variation of approximately 10% of the mean value, the proposed method can reconstruct the spectrum from approximately 1/20 of the number of measurement points in the Fourier basis [11,17,18,19,20,46,50]. In terms of noise tolerance, when the SNR is 7 or higher, the method can reconstruct a spectrum if the number of measurement points is within the theoretically guaranteed range; however, when the SNR is 0.4, the performance deteriorates drastically.
- An evaluation of the proposed method on a simulated vibration signal of a broken gear demonstrated that it is possible to reconstruct the vibration with an MSE of less than 2% from several measurement points (α = 0.029), which is approximately 1/35 of the full sampling. In addition, the method can clearly detect sidebands originating from gear eccentricity, which could not be observed in the Fourier basis owing to the effect of rotational speed fluctuation and the identification in the order domain.
- The results of the propeller drive experiment showed that the proposed method enables CS with an MSE of 8.5% even with the number of points amounting to approximately 1/43 of full sampling (α = 0.023). In addition, the findings suggest that fault diagnosis can be performed by monitoring the increase in the rotation frequency component and decrease in the propeller passing frequency component in the order spectrum measured using the proposed method.
- The experiments conducted on a bearing with a defective outer ring showed that the proposed method can perform the CS of a portion of the anomalous vibration caused by an outer ring defect with the number of points (α = 0.039) amounting to approximately 1/26 of the full sampling. By applying envelope processing and Fourier transform to the signal reconstructed via CS, components consistent with the BPFO can be extracted. Therefore, fault detection is concluded to be possible; however, the reconstruction error is large and the MSE is greater than 60%. This is possibly because the vibration caused by the outer ring defect and the natural vibration of the bearing are shock waveforms with many components in the 1500–2000 Hz range and damped vibration with a wide peak bandwidth, which are not sparse in the order basis.

These results show that CS using the order basis can achieve an improved signal compression ratio, enabling the diagnosis of gear, bearing, and propeller failures. However, the compression performance deteriorated significantly when shock-damped waveforms, such as bearing outer ring defects, were measured. Our future research will explore developing a basis for effective observation of such waveforms. The developed CS with an order basis is expected to be useful in the fields of structural health monitoring and vibration measurement.

## Figures and Tables

**Table 1:** Condition of Fourier and order basis.

Basis Type	Fourier	Order
Lower frequency limit: *f_bl_*	0 [Hz]	0 [-]
Upper frequency limit: *f_bh_*	*f_s_*/2 [Hz]	*f_s_*$/(2\;\bar{f_{r}}$) [-]
Length of basis: *n*	*f_s_ T_s_*	

**Table 2:** Bearing specifications.

Outer Diameter: *D_out_*	Inside Diameter: *D_in_*	Rolling Element Diameter: *d*	Pitch Circle Diameter: *D_p_*	Contact Angle: *θ_c_*
32 mm	15 mm	4.76 mm	23.5 mm	30°

## Data Availability

Data will be made available upon request.
